# Enhanced Degradation of Juvenile Hormone Promotes Reproductive Diapause in the Predatory Ladybeetle *Coccinella Septempunctata*

**DOI:** 10.3389/fphys.2022.877153

**Published:** 2022-04-29

**Authors:** Yu-Yan Li, Jun-Jie Chen, Meng-Yao Liu, Wei-Wei He, Julie A Reynolds, Ya-Nan Wang, Meng-Qing Wang, Li-Sheng Zhang

**Affiliations:** ^1^ Key Laboratory of Natural Enemy Insects, Ministry of Agriculture and Rural Affairs, Institute of Plant Protection, Chinese Academy of Agricultural Sciences, Beijing, China; ^2^ Department of Evolution, Ecology, and Evolutionary Biology, The Ohio State University, Columbus, OH, United States

**Keywords:** juvenile hormone degradation genes, reproductive diapause, ovarian arrest, lipid accumulation, juvenile hormone analogue, diapause manipulation

## Abstract

Improved knowledge on the regulation of reproductive diapause in *Coccinella septempunctata*, an important predator of aphids, is crucial for improving shelf-life and mass production of the ladybeetles. In many insects, the absence of juvenile hormone (JH) is a central regulator of reproductive diapause. JH is principally degraded by JH esterase (JHE) and JH epoxide hydrolase (JHEH). Previous studies have shown that genes encoding these enzymes were upregulated in early diapause of *C. septempunctata,* but whether increased JH degradation contributes to the reduction of JH levels and facilitates reproductive diapause remains unknown. Here, we investigate the role of JH and JH degradation genes during reproductive diapause in *C. septempunctata* females. Applying methoprene, a JH analogue, to the diapause preparation females clearly elevated JH signaling and reversed diapause program, suggesting that a lower level of JH is critical for the induction of reproductive diapause in the ladybeetle. Full-length cDNA sequences of *JHE* and *JHEH* were cloned and characterized, and their deduced proteins contain all the conserved active domains and typical motifs as identified in other insects. The expressions of *JHE* and *JHEH* were both significantly increased in diapause preparation and remained at a high level for a period throughout diapause, and then decreased after the termination of diapause. Knocking down these JH degradation genes clearly increased the expression levels of JH-inducible genes *Krüppel-homolog 1* (*Kr-h1*) and *vitellogenin* (*Vg*), indicating an elevated JH level. Simultaneously, silencing JH degradation genes distinctly reduced diapause-related features and promotes reproduction, indicated by accelerated ovary growth, yolk deposition, and suppressed lipid accumulation. These results indicate that the enhanced JH degradation plays a critical role in regulating reproductive diapause of *C. septempunctata*.

## Introduction

A tremendous adaptative strategy in the life cycle of many insects is that they can enter diapause, an arrested developmental state, to survive periods of unfavorable environmental conditions such as low temperature, desiccation and food absence (Tauber et al., 1986; Danks, 1987). Diapause is an environmentally preprogrammed and hormonally mediated response that occurs at a specific stage for many insect species (Denlinger, 2002; Denlinger et al., 2012). It is a dynamic physiological process consisting of several successive phases, and each phase of diapause involves specific changes in physiological, hormonal and molecular systems (Koštál, 2006; Hand et al., 2016; Koštál et al., 2017; Reynolds, 2017). Diapause is frequently characterized by developmental suppression, accumulation of energy reserves, metabolic depression, increased stress-resistant, and extended lifespan (Denlinger, 2000; Hahn and Denlinger, 2011; Hand et al., 2016). From the applied point of view, manipulation diapause may improve long-term storage, shipment, mass rearing and effective application of beneficial insects (Denlinger, 2008; Tougeron, 2019). This has been validated in mass production of several biological control agents, including *Harmonia axyridis* (Ruan et al., 2011), *Trichogramma dendrolimi* (Zhang et al., 2018), *Microplitis mediator* (Hun et al., 2005) and *Chrysopa formosa* (Li et al., 2018). Thus, an improved knowledge of diapause is not only essential for understanding mechanisms by which insects adapt to environmental changes, but also for the development of biological control.

The ladybeetle, *Coccinella septempunctata* (Linnaeus), is an economically important predator primarily used to control aphids and other crop pests such as whiteflies, mites, thrips and lepidopteran pests (Deligeorgidis et al., 2005; Hodek and Michaud, 2008). This predator has been commercially mass-cultured and widely used in greenhouses and croplands in Europe, Asia and North America (Van Lenteren, 2012; Zhou H. et al., 2014; Yang et al., 2014). Adults of *C. septempunctata* are capable of entering diapause in summer or winter, depending on geographical distribution of different populations (Katsoyannos et al., 1997; Ren et al., 2016). In northern China, *C. septempunctata* adults enter winter diapause in response to short photoperiods and low temperatures (Wang et al., 2013). Our previous work indicates that the diapausing ladybeetles can be stored at 18°C for 120 days with no obvious decrease in their fitness, implying that diapause could be exploited to enhance their shelf-life and mass rearing (Wang, 2012). During winter diapause of *C. septempunctata*, arrested ovarian development, vitellogenesis inhibition, and increased lipid accumulation are the most obvious phenotypes observed in female adults (Ren et al., 2016). We also found a conspicuous series of changes at the proteomic and transcriptomic levels in diapausing females, and we identified a number of specifically expressed genes and proteins involved in lipid storage, energy metabolism and hormonal signaling pathways (Qi et al., 2015; Ren et al., 2016). The diapause-related functions of several genes involved in lipid accumulation have been revealed in *C. septempunctata* (Xiang et al., 2021), but little is known about the hormonal and molecular regulatory mechanisms that generate reproductive diapause phenotypes in this ladybeetle.

Juvenile hormones (JHs), belong to a family of sesquiterpenoids synthesized primarily by the corpora allata (CA), and they regulate a wide spectrum of critical biological events in insects, including development, metamorphosis, reproduction and diapause (Denlinger et al., 2012; Jindra et al., 2013; Denlinger and Armbruster, 2014). In most insect species, the absence of juvenile hormone (JH) is recognized as a central regulator during reproductive diapause (Denlinger et al., 2012). Thus, accurate regulation of JH levels is crucial for precisely directing the reproductive diapause process. JH levels are balanced by regulated biosynthesis and degradation in insect hemolymph (Supplementary Figure S1). The synthesis of JH in the CA is under complex control by various neuroendocrine and neuronal factors (Kang et al., 2014). JH in the hemolymph and the tissues is mainly degraded by three enzymes, including JH esterase (JHE), JH epoxide hydrolase (JHEH) and JH diol kinase (JHDK) (Zhang et al., 2005) (Supplementary Figure S1). In many instances of reproductive diapause, a marked downregulation of genes encoding enzymes involved in JH biosynthesis is accompanied with the upregulation of genes involved in JH metabolism, suggesting that the JH titers during diapause are collaboratively regulated by the JH biosynthesis and degradation pathways (Chen H. et al., 2021; Ma et al., 2021a; Gao et al., 2021; Wu et al., 2021).

The shutdown of JH biosynthesis has been demonstrated to be critical in triggering the initiation of reproductive diapause in several species, including *Colaphellus bowringi*, *Harmonia axyridis*, and *Culex pipiens* (Sim et al., 2015; Gao et al., 2021; Tian et al., 2021). There is also some evidence that JH degradation has a role in regulating diapause. In *C. bowringi* and *H. axyridis*, JH degradation facilitates lipid storage and stress tolerance during diapause preparation (Guo et al., 2019; Gao et al., 2021). In addition, a previous study from our lab indicated that the transcript abundances of the genes encoding JHE and JHEH were both significantly increased in early diapause and decreased after diapause termination in female adults of *C. septempunctata* (Qi et al., 2015). How changes in these genes influences diapause in *C. septempunctata* remains unknown. A robust negative relationship between JH titers and the activity or transcriptional expression of JHE and JHEH has been reported in *Anthonomus grandi*s (Taub-Montemayor et al., 1997), *Leptinotarsa decemlineata* (Vermunt et al., 1999), and *H. axyridis* (Gao et al., 2021), suggesting the JH degradation enzymes play major roles in the breakdown of JH and the regulation of JH levels. Several recent studies revealed that silencing *JHE* or *JHEH* in diapause-destined adults increased JH signaling and reversed some diapause-related traits, evidencing that the two JH degradation enzymes are necessary for maintaining diapause phenotypes (Guo et al., 2019; Gao et al., 2021). Taken together, we hypothesize that the upregulation of *JHE* and *JHEH* may promote reproductive diapause features by reducing JH levels in diapausing females of *C. septempunctata*.

In this study we test the hypothesis that a diapause in females of *C. septempunctata* is due to a reduction in JH titers, and upregulation of JHE and JHEH contribute to the decrease of JH level. First we evaluated the role of JH in reproductive diapause by testing the responsiveness of diapause-destined females to methoprene, a JH analog (JHA). We then cloned and identified the full-length sequences of *CsJHE* and *CsJHEH*, and we measured the transcript levels of these two genes at different phases during diapause processes. We also evaluated the function of *JHE* and *JHEH* in reproductive diapause using RNA interference (RNAi)-mediated loss-of-function experiments. We found that JHA enhances JH signaling and reduces diapause-related traits. Knockdown of JH degradation genes in diapause-destined females blocks diapause. Taken together, the experiments we present underscore the critical role of JH degradation in promoting reproductive diapause, and they suggest techniques that could be exploited to improve the shelf-life and effective release of mass-produced predatory ladybeetles.

## Materials and Methods

### Insect Rearing and Sample Preparation

A colony of *C. septempunctata* was maintained in the laboratory as described (Qi et al., 2015; Xiang et al., 2021). Ladybeetles were fed aphids (*Aphis glycines* Matsumura). Normal developmental conditions were 24 ± 1°C, with a long-day photoperiod of (16 h light: 8 h dark per 24 h), and relative humidity (RH) of 70 ± 10%. Diapausing adults were generated by placing newly emerged adults (within 24 h after eclosion) in diapause-inducing conditions of 18 ± 1°C, with a short-day photoperiod of (10 h light: 14 h dark per 24 h) and RH 70 ± 10%. Adults enter diapause approximately 30 days after being placed in diapause-inducing conditions. Post-diapause adults (PD) were produced by transferring diapause 10 days adults (40 days after transferring to diapause-inducing conditions) to normal developmental conditions until first oviposition. To evaluate relative mRNA expression at different stages we sampled the newly emerged, nondiapausing female adults (NE); nondiapausing, 30 days-old female adults of *C. septempunctata* (ND); diapausing female adults at the pre-diapause stage (10 days and 20 days after transferring to diapause-inducing conditions; D10 and D20, respectively); diapause stage (30 days and 60 days after transferring to diapause-inducing conditions; D30 and D60, respectively); and post-diapause female adults (PD). The timeline for insect sampling was shown in Supplementary Figure S2. All insects were collected, cleaned and frozen with liquid nitrogen. All the samples were stored at a refrigerator (−80°C) until analysis. Each treatment was performed with three biological replicates, and each replicate contains one female.

### Molecular Cloning

Total RNA was extracted from one diapausing female adult of *C. septempunctata* using RNAiso Plus reagent Method (TaKaRa, Dalian, China). The purity and quantity of isolated RNA were assessed using a P-class Nanophotometer (Implen, Germany), and the RNA integrity was determined by 1.5% agarose gel electrophoresis. First strand cDNA was synthesized from total RNA using a Goldenstar RT6 cDNA Synthesis Kit (TSINGKE, Beijing, China) according to the manufacture’s protocol. Initial sequence fragments of *JHE* and *JHEH* genes were identified from the transcriptome data of *C. septempunctata* (GSE75645). Specific primers for amplifying full-length coding regions of these genes were designed (Supplementary Table S1) and synthesized by TSINGKE Biological Technology Company (Beijing, China). Sequences were confirmed from cloned PCR products. PCR was performed with I-5 2X High-Fidelity Master Mix (TSINGKE). PCR products were separated on a 1.5% agarose gel and purified using NucleoSpin Gel and PCR Clean-up kits (Macherey-Nagel, Duren, Germany). Cleaned PCR products were ligated into a pMD18-T Vector (TSINGKE), transformed into DH5α Chemically Competent Cells (TSINGKE), and then sequenced (Sangon Biotech, Shanghai, China). Full-length cDNA was obtained by rapid amplification of cDNA ends (RACE), performed with the SMARTer RACE 5'/3' Kit (Clontech, CA, Untied States), following the manufacture’s protocol. The amplification PCR products were purified as described above. PCR products of the expected size were excised from the gels, cloned and transformed using the In-Fusion HD Cloning Kit (Clontech) and then sequenced.

### Sequence and Phylogenetic Analysis

The amino acid sequences were deduced with the ExPASy Translate tool (Gasteiger et al., 2003)^1^ and physiochemical features of the proteins were predicted using ExPASy Proteomics Server (Gasteiger et al., 2003). The conserved domain analysis was performed using the CDD Search program (Lu et al., 2020)^2^. Putative signal peptide was discovered with SignalP server (Teufel et al., 2022)^3^. The across-membrane structure was predicted at TMHMM Server v.2.0 (Möller et al., 2001)^4^. To infer the evolutionary history of *C. septempunctata JHE* and *JHEH*, JHE and JHEH protein sequences of various species were downloaded from the National Center for Biotechnology Information (NCBI) web server (Sayers et al., 2021)^5^ and aligned with sequences of CsJHE and CsJHEH using ClustalW 2 and ESPript 3.0 web server (Gouet et al., 1999)^6^. A phylogenetic tree was constructed by neighbor-joining method in MEGA v6.0 software, and bootstrap analysis was preformed using 1,000 replicates.

### Expression Profiles of Genes Determined by qRT-PCR

Quantitative real-time PCR (qRT-PCR) was used to measure changes in transcript abundance of *CsJHE* and *CsJHEH* in nondiapausing and diapausing adult females. Expression levels of *Krüppel-homolog 1* (*Kr-h1*), *vitellogenin* (*Vg*) and *fatty acid synthase* (*Fas1* and *Fas2*) were measured following JHA treatment and RNAi to evaluate the effect of those treatments.

Briefly, following total RNA extraction, the first-strand cDNA was synthesized using the PrimeScript Reagent Kit with gDNA Eraser (Perfect Real Time, TaKaRa). The qRT-PCR reactions were performed using TOROGreen^®^ 5G qPCR Premix (Toroid Technology Limited). All reactions were performed in triplicate in a total volume of 20 μl containing 10 μl TOROGreen Premix, 0.8 μl of each primer, 1 μl sample cDNA, and 7.4 μl nuclease-free water. The protocol was as follows: initial preincubation at 95°C for 5 min, followed by 40 cycles at 98°C for 10 s and 56°C for 20 s. At the end of the reaction, a final melting curve analysis was conducted from 65 to 97°C to ensure the specificity of each primer pair. The primer sequences used for qRT-PCR are listed in Supplementary Table S1. Efficiency of the primers for each gene was previously evaluated by serial dilutions of cDNA pooled from the samples. Relative expression levels of target genes were calculated by the 2^−ΔΔCT^ method using actin and 18S as internal references (Qi et al., 2015; Xiang et al., 2021).

### JHA Treatment

To determine the role of JH in regulation of reproductive diapause in *C. septempunctata*, methoprene (JHA) was applied to newly emerged female adults 2 days after transfer to diapause-inducing conditions. Briefly, methoprene (Sigma, St. Louis, MO, United States) was dissolved in acetone and diluted to a concentration of 100 μg/μl. One microliter (100 μg) of the methoprene solution was dropped onto the pronota of female adults. An acetone solution was used as a negative control, and untreated females as blank controls. Following JHA application, females were reared continually under diapause-inducing conditions. Total lipid content (see below), visual changes in ovariole length and the state of lipid storage (see below), and mRNA expression of *Vg*, *Kr-h1*, *CsJHE, CsJHEH, Fas1,* and *Fas2* were evaluated 11 days after JHA treatment.

### Total Lipid Content

Total lipid content in 10 females in each treatment was measured using the vanillin assay previously described by Xiang et al. (2021). Briefly, the fresh mass (FM) of each female was measured before drying at 60°C for 48 h, after which the initial dry mass (DM1) was weighed. Subsequently, lipids were extracted twice with a 1 ml chloroform and methanol (2:1) solution, and then reweighed to determine the dry mass (DM2) after drying at 60°C for 48 h. The stored lipid content was determined by subtracting DW2 from DW1, and the total lipid content was expressed as the percentage of stored lipid content (DW1-DW2) to the initial dry mass (DW1).

### Visual Changes in Ovarian Development and Lipid Storage

Ovarian development, ovariole length, and the state of lipid storage were evaluated on day 11 following JHA treatment and RNAi using a Leica VHX-2000 stereomicroscope (Leica Microsystems, Wetzlar, Germany). The ovarian developmental state of females in JHA treatment and dsRNA-injected groups were observed and photographed by a VH-Z20W zoom lens with ×50 magnification. For females in the Control, Acetone treatment and ds*GFP*-injected groups, a camera lens with ×100 magnification was used for taking photos. The state of lipid storage of females in all treatment groups were photographed by a zoom lens with ×50 magnification. For each treatment, ten females were used for measurement.

### RNA Interference

To evaluate the function of *CsJHE* and *CsJHEH* during diapause development of *C. septempunctata*, RNAi was performed using the Green fluorescent protein (GFP) as a negative control. The target genes *CsJHE*, *CsJHEH* and *CsGFP* were first amplified using specific primers including a T7 promoter sequence (Supplementary Table S1). PCR products were purified and used as template for dsRNA synthesis using a MEGAscript T7 High Yield Transcription Kit (Invitrogen) following the manufacturer’s protocol. The quality of dsRNAs were checked by agarose gel electrophoresis (1.5%), and the quantity was determined by a Nophotometer P-class (Implen, Germany). Finally, each dsRNA was dissolved in RNase-free water to a concentration of 2 μg/μl and stored at −80°C until use.

One microliter dsRNA (2 μg) was injected into the abdomen of each female adult that reared under diapause inducing conditions for 2 days (3 days after eclosion) with a microinjector (NK2, Eppendorf, Germany). Females injected with dsRNA were reared in pairs under diapause-induction conditions. Three biological replicates were performed with 100 female adults per replicate. The efficiency of RNAi was checked at 11 days after injection, and the relative mRNA expression levels of *JHE* and *JHEH* were examined by qRT-PCR.

To evaluate whether silencing of *JHE* and *JHEH* could reduce the reproductive diapause features, we measured expression of *Kr-h1*, *JHE*, *JHEH*, *Vg*, *Fas1* and *Fas2*; total lipid content; visual changes in lipid storage, the ovary developmental morphology and ovariole length on day 11 after injection of dsRNA.

### Statistical Analysis

All statistical analyses were performed in GraphPad Prism 7.0 (GraphPad Software, San Diego, CA, Untited States). One-way ANOVA followed by Tukey’s test (*p* = 0.05) was performed on mRNA expression data and total lipid content to identify differences among different developmental stages. Differences in gene expression, ovariole length, and lipid content between the treatment and control was evaluated using Student’s *t*-test. All values are expressed as means ± standard deviation (SD).

## Results

### Cloning and Sequence Analysis of JH Degradation Genes

**FIGURE 1:**
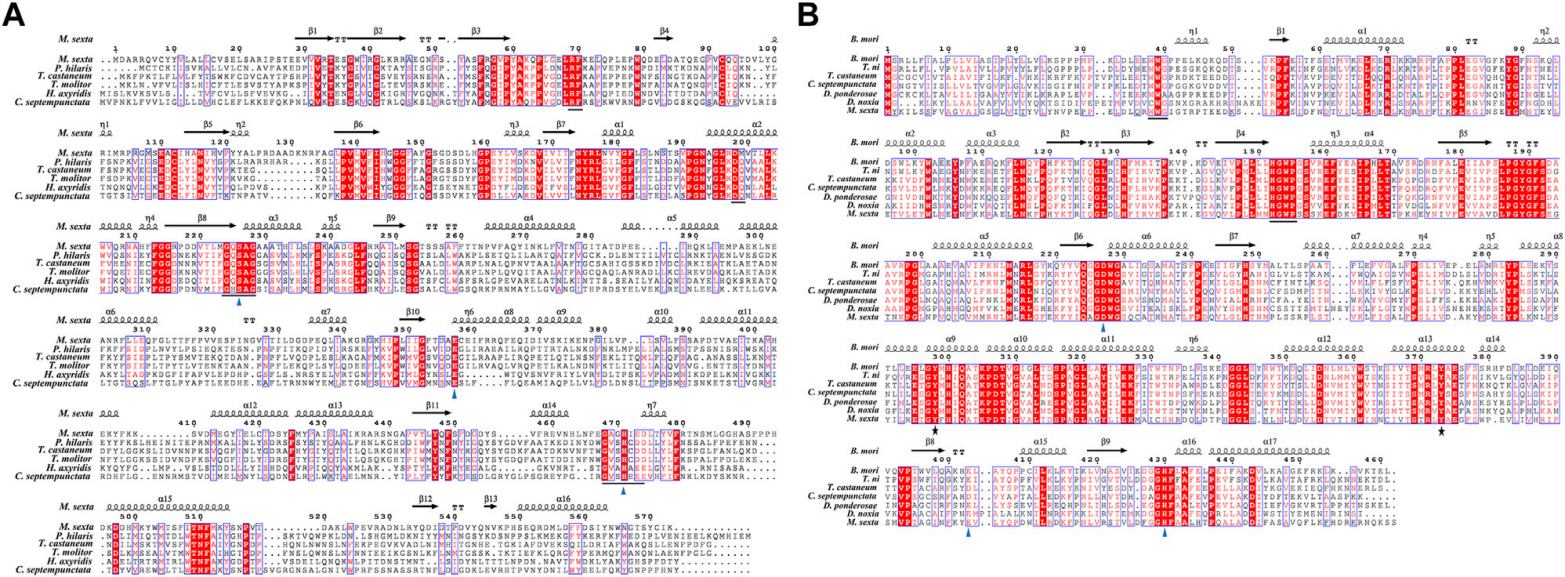
Multiple-sequence alignment of *C. septempunctata* JHE **(A)** and JHEH **(B)** with the corresponding proteins from other insect species. Alpha-helices, eta-helices, beta strands and beta turns are marked by α, η, β and TT, respectively. **(A)** The predicted protein of *Cs*JHE contains five highly conserved motifs ‘RF’, ‘DQ’, ‘GxSxG’, ‘E’, and ‘GxxHxxD/E’, as underlined in Panel A. The catalytic triad residues (Ser-223, His-478 and Glu-358) is labeled with triangles. **(B)** The N-terminal membrane anchor motif ‘WWG’ and the conserved ‘HGWP’ motif in *Cs*JHEH are underlined; the catalytic triad (Asp228, Asp404, and His430) are marked as triangles, and two tyrosine residues (Tyr298 and Tyr373) are labeled with stars. **(A)** The JHE protein sequences used with the GenBank accession numbers are listed in the order illustrated: *Manduca sexta* (AAG42021.2), *Psacothea hilaris* (BAE94685.1), *Tribolium castaneum* (NP_001180223.1), *Tenebrio molitor* (AAL41023.1), *Harmonia axyridis* (BAE16976.1), and *Coccinella septempunctata* (AND78201.1). **(B)** The JHEH protein sequences followed by the GenBank accession numbers are listed in the order illustrated: *Bombyx mori* (AAQ87024.1), *Trichoplusia ni* (AAB18243.1), *Tribolium castaneum* (EFA00568.1), *Coccinella septempunctata* (AZB52850.1), *Dendroctonus ponderosae* (XP_019766997.1), *Diuraphis noxia* (XP_015364968.1), and *Manduca sexta* (AAC47018.1).

We cloned the genes encoding *Cs*JHE and *Cs*JHEH, two key enzymes involved in JH degradation, from *C. septempunctata*, and we deposited the full-length sequences to GenBank with the accession number of KX013559 and MH932586. The cDNA sequence of *CsJHE* is 2,317 bp in length, containing an ORF of 1,755 bp. It encodes a protein of 584 amino acid residues with a predicted molecular mass of 66.46 kDa and a theoretical isoelectric point of 8.36. A carboxylesterase family conserved domain exists between residues 30 and 560 of the deduced *Cs*JHE protein. The *Cs*JHE protein also contains a putative signal peptide of 19 amino acids at the N-terminus, suggesting JHE may be a secretory protein. By aligning *Cs*JHE protein sequence with orthologs from different insect species, we found that *Cs*JHE contains five highly conserved motifs (RF, DQ, GxSxG, E, and GxxHxxD/E), including the catalytic triad residues (Ser-223, His-478 and Glu-358) (Figure 1A).

**FIGURE 2:**
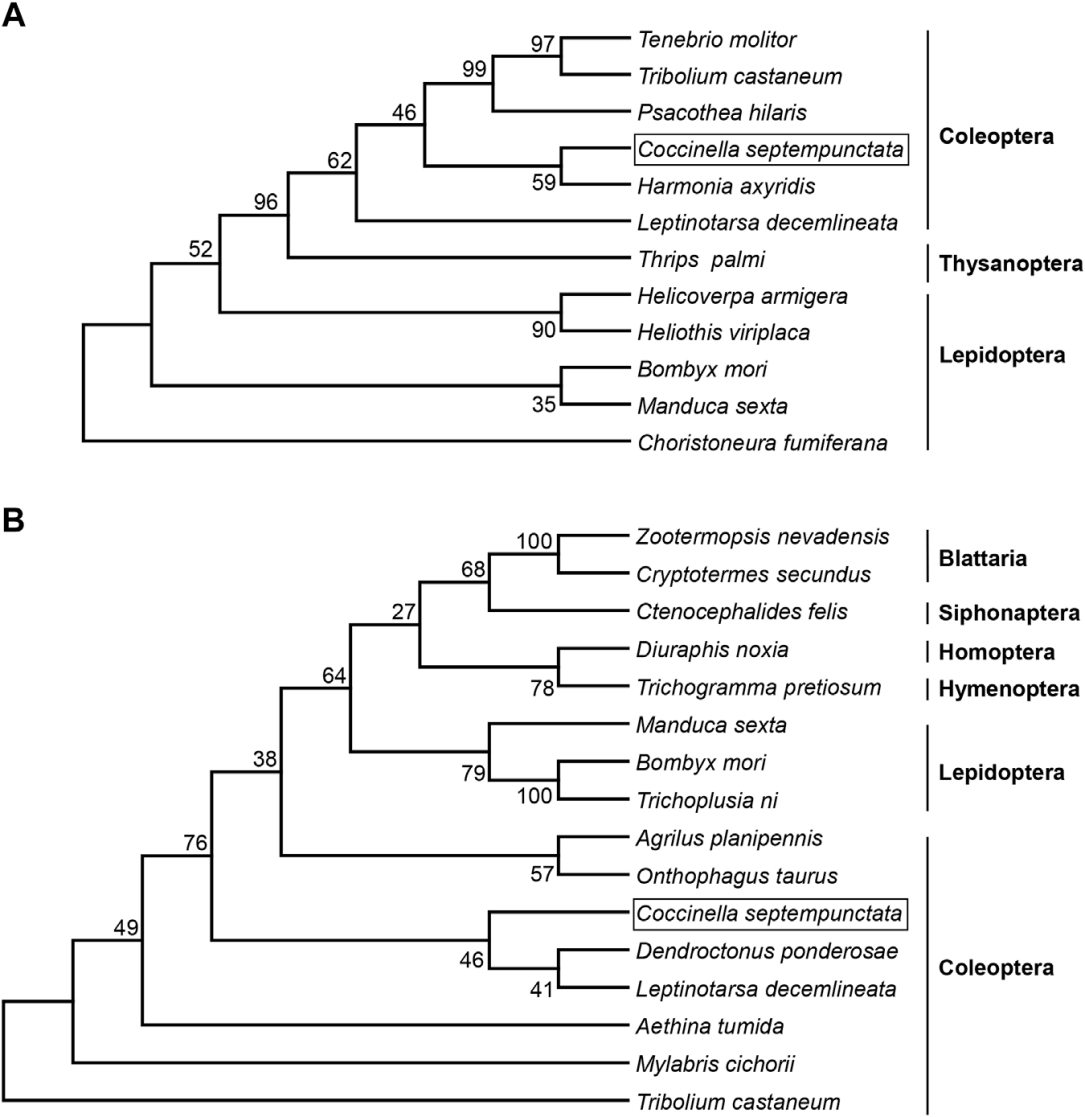
Evolutionary relationship of JHE and JHEH from *C. septempunctata* and other insects. A phylogenetic tree was constructed based on amino acid sequences of JHE **(A)** and JHEH **(B)** from different insect species using the neighbor-joining method with 1,000 bootstraps. The numbers at the forks indicates the bootstrap. **(A)** The protein sequences of JHE used for phylogenetic analysis were as follows: *Tenebrio molitor* (AAL41023.1), *Tribolium castaneum* (NP_001180223.1), *Psacothea hilaris* (BAE94685.1), *Coccinella septempunctata* (AND78201.1), *Harmonia axyridis* (BAE16976.1), *Leptinotarsa decemlineata* (AGC95172.1), *Thrips palmi* (XP_034246354.1), *Helicoverpa armigera* (AEB77712.1), *Heliothis viriplaca* (AGB93712.1), *Bombyx mori* (AAR37335.1), *Manduca sexta* (AAG42021.2), and *Choristoneura fumiferana* (AAD34172.1). **(B)** The JHEH protein sequences used for phylogenetic analysis were as follows: *Zootermopsis nevadensis* (KDR10172.1), *Cryptotermes secundus* (XP_023724762.1), *Ctenocephalides felis* (XP_026476717.1), *Diuraphis noxia* (XP_015364968.1), *Trichogramma pretiosum* (XP_014233585.1), *Manduca sexta* (AAC47018.1), *Bombyx mori* (AAQ87024.1), *Trichoplusia ni* (AAB18243.1), *Agrilus planipennis* (XP_025832013.1), *Onthophagus taurus* (XP_022920508.1), *Coccinella septempunctata* (AZB52850.1), *Dendroctonus ponderosae* (XP_019766997.1), *Leptinotarsa decemlineata* (AKF11871.1), *Aethina tumida* (XP_019870196.1), *Mylabris cichorii* (AMR44689.1), and *Tribolium castaneum* (EFA00568.1).

Full length cDNA sequence of *CsJHEH* is 2,077 bp long, comprising an ORF of 1,380 bp, and encodes a protein of 459 amino acids. This predicted protein of *Cs*JHEH had a molecular weight of 51.39 kDa and a theoretical isoelectric point of 8.79. Two typical conserved domains, including epoxide hydrolase N-terminus (EHN) (residues 50–149) and alpha/beta hydrolase (Abhydrolase) fold domain (residues 146–330), which are responsible for the basic catalytic activity of epoxide hydrolase were identified in *Cs*JHEH. *Cs*JHEH contains a predicted N-terminal transmembrane helical structure located at amino acid residues 7–29, which indicates that *Cs*JHEH is a transmembrane protein. There is also a putative signal peptide of 26 amino acids at the N-terminus in *Cs*JHEH, suggesting that JHEH may be secreted into extracellular space. Multiple sequence alignment of *Cs*JHEH showed that it contains two conserved ‘WWG’ and ‘HGWP’ motifs, a catalytic triad (Asp228, Asp404, and His430), and two tyrosine residues (Tyr298 and Tyr373) (Figure 1B). Among the motifs, the N-terminal motif ‘XWG’ (where X is an aromatic residue) is predicted to be a membrane anchor for the localization of mEHs to the endoplasmic reticulum. This motif is highly conserved in JHEH of *C. septempunctata* and other insect species (Zhou K. et al., 2014). The motif ‘HGWP’ is proposed to facilitate the catalysis of JHEH, and the two tyrosine residues is predicted to stabilize and donate protons to the oxygen atom of expoxide ring. To investigate the phylogenetic relationship of JHE and JHEH protein from *C. septempunctata* and the other insect species, a phylogenetic tree was constructed. The results showed that CsJHE and CsJHEH were both classified within the Coleoptera branch, with CsJHE is more closely related to JHE from *Harmonia axyridis* (Figure 2A), and CsJHEH is clustered with two JHEHs from *Dendroctonus ponderosae* and *Leptinotarsa decemlineata* (Figure 2B).

### Expression Profiles of *CsJHE* and *CsJHEH* Throughout Diapause

**FIGURE 3:**
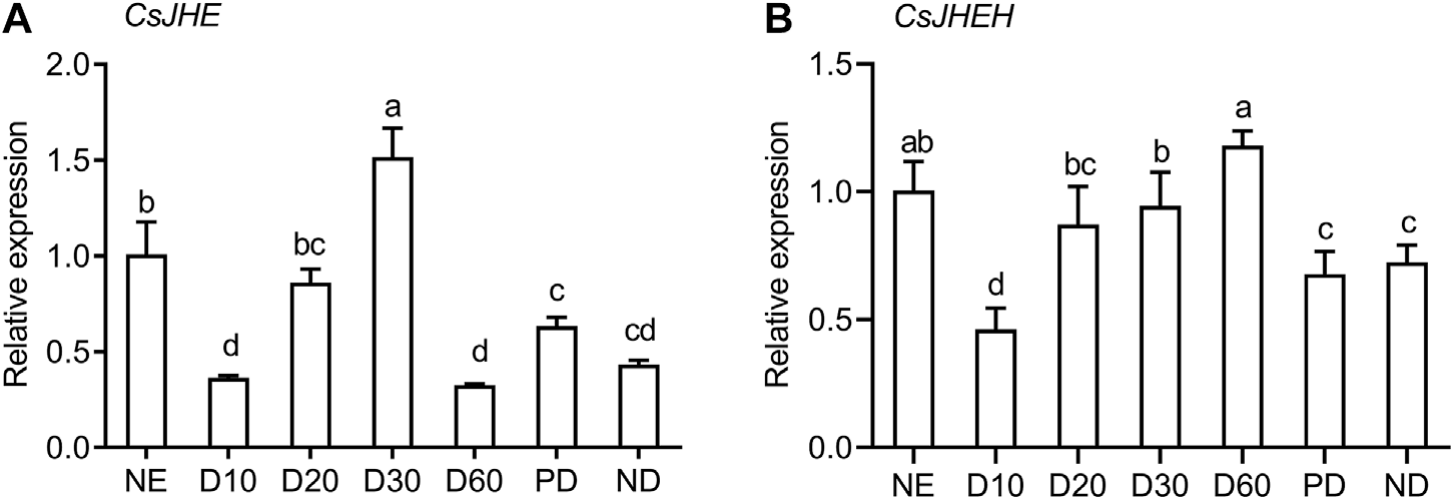
JH degradation genes are highly expressed during reproductive diapause in females of *C. septempunctata*. Relative mRNA abundance of JHE **(A)** and JHEH **(B)** in nondiapausing (NE and ND), diapausing (females induced diapause for 10, 20, 30, and 60 days) and post-diapausing (PD) female adults were measured with RT-qPCR. Each point represents the mean relative mRNA expression of 3 biological replicates, and bars represent the standard deviation. Different letters indicate significant differences (*p* < 0.05), as determined by one-way ANOVA followed by Tukey’s multiple comparison test.

We investigated the transcriptional profiles of *JHE* and *JHEH* at different diapause stages in *C. septempunctata*, including the prediapause stage (D10 and D20), the diapause stage (D30 and D60) and the post-diapause stage (PD) (Figure 3). In the early diapause induction phase (D10), the mRNA abundance of *JHE* and *JHEH* were both at the lowest level, which is close with their expression in nondiapause individuals (ND) and in post-diapause females. During the preparation stage for diapause, the transcript levels of both genes significantly increased from day 10–30. The expression of *JHE* peaked in the early diapause (D30) (Figure 3A). Of special interest, *JHE* expression decreased abruptly from day 30 to day 60, and at day 60 was at levels comparable to day 10 diapausing adults and nondiapausing adults. After diapause termination, the expression of *JHE* was up-regulated slightly. The highest level of *JHEH* expression occurred 60 days (D60) post-diapause induction (i.e., approximately 30 days after diapause entry) (Figure 3B). Expression of *JHEH* in the post-diapause stage was similar to that of nondiapausing females. Additionally, we noted that *JHE* and *JHEH* were expressed at relatively higher levels in the newly emerged adults, but transcript abundance dropped significantly to a lower level at day 10 following diapause induction.

### Silencing JH Degradation Genes Reduces Diapause-Related Features

**FIGURE 4:**
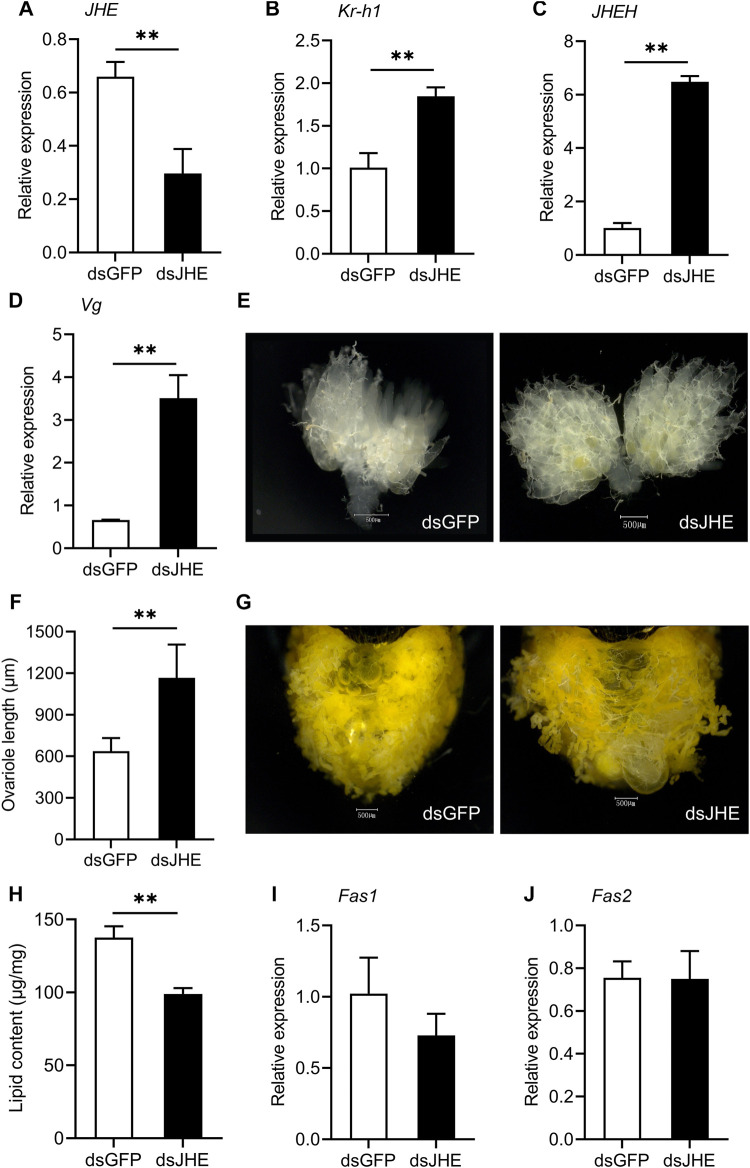
Knockdown of *JHE* in the diapause-destined females promotes ovary development and yolk deposition, but reduces lipid accumulation. Relative expressions of *JHE* **(A)**, *Kr-h1* **(B)**, *JHEH* **(C)** and *Vg* **(D)**, yolk deposition **(E)**, ovariole length **(F)**, lipid accumulation status **(G)**, lipid content **(H)** and the expression levels of *Fas1* **(I)** and *Fas2* **(J)** were determined on the 11th day after injection of dsRNA. Ovarian developmental state of females in ds*GFP* control group were photographed by a VH-Z20W zoom lens with ×100 magnification, and a ×50 magnification of camera lens was used for photographing ovary development of ds*JHE*-injected females and lipid storage of females in both two groups. Values are means ± SD (*n* = 3–10). Asterisks (*) represent significant differences between the ds*GFP* control and ds*JHE* treatment (Student’s *t*-test; *p < 0.05, **p < 0.01).

To investigate the function of JH degradation during reproductive diapause, we used RNAi to silence *JHE* and *JHEH* in females preparing to enter diapause. Injecting *JHE* dsRNA into diapause-destined female adults significantly decreased the expression levels of *JHE* compared to the ds*GFP* control. *JHE* mRNA abundance was reduced by 55.1% on the 11th day post dsRNA injection (Figure 4A). Knocking down *JHE* significantly increased the relative expression of *Kr-h1* and *JHEH* in the females destined for diapause (Figures 4B,C). Silencing *JHE* also promoted ovary development, as evidenced by a distinct upregulation of *Vg* mRNA abundance, signs of yolk deposition in oocytes and increased variole length (Figures 4D–F). As expected, depletion of *JHE* did cause a marked reduction of the lipid accumulation in the diapause-programmed females (Figures 4G,H), but there was no significant difference for the transcriptional levels of *Fas1* and *Fas2* (Figures 4I,J).

**FIGURE 5:**
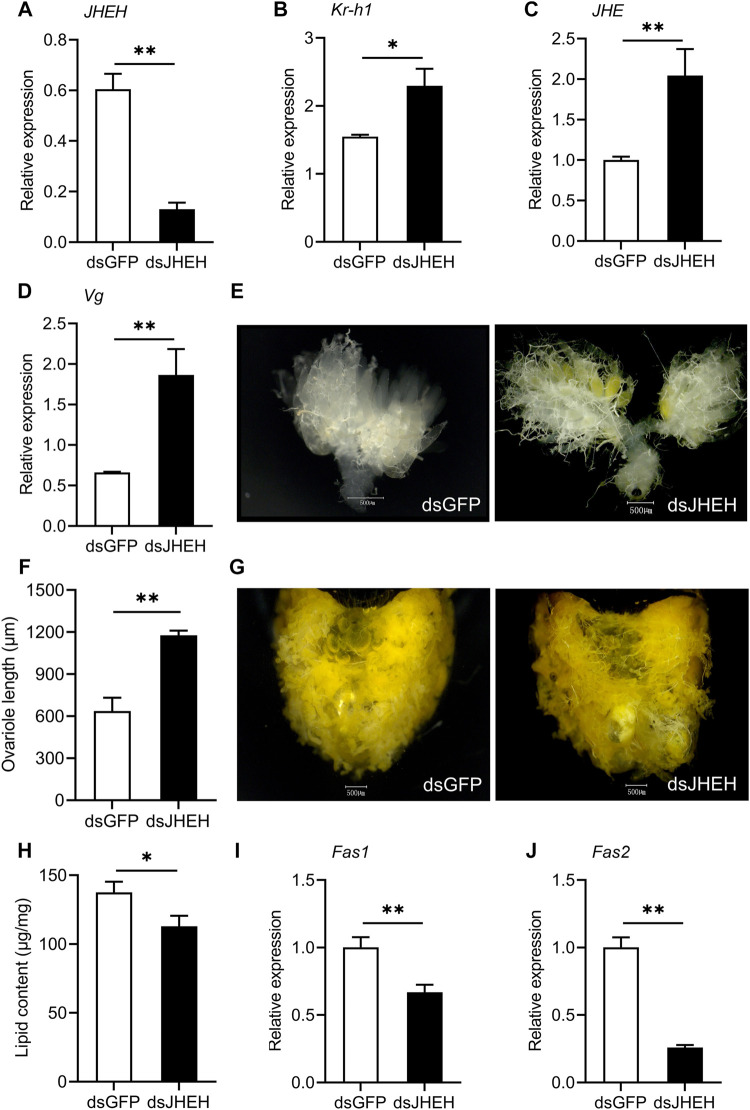
Depletion of *JHEH* in the diapause-destined females accelerates ovarian growth and yolk deposition, whereas suppresses lipid storage. Relative mRNA abundances of *JHEH* **(A)**, *Kr-h1* **(B)**, *JHE* **(C)** and *Vg* **(D)**, yolk deposition **(E)**, ovariole length **(F)**, lipid storage state **(G)**, lipid content **(H)** and the expressions of *Fas1* **(I)** and *Fas2* **(J)** were measured at 11 days after dsRNA injection. Ovarian developmental state of females in ds*GFP* control group were photographed by a VH-Z20W zoom lens with ×100 magnification, and a ×50 magnification of camera lens was used for photographing ovary development of ds*JHEH*-injected females and lipid storage of females in both two groups. Values are means ± SD (*n* = 3–10). Asterisks (*) represent significant differences between the ds*GFP* control and ds*JHEH* treatment (Student’s *t*-test; *p < 0.05, **p < 0.01).

The expression level of *JHEH* in dsRNA-injected females was strongly reduced compared with the ds*GFP* control (Figure 5A). As seen with knockdown of ds*JHE*, depletion of *JHEH* resulted in a distinct upregulation of *Kr-h1* and *JHE*, suggesting that both *JHE* and *JHEH* suppress JH signaling in females during diapause preparation (Figures 5B,C). Similar to the results caused by the knockdown of *JHE*, silencing *JHEH* had a pronounced effect on promoting ovarian development and yolk deposition (Figures 5D–F). However, *JHEH* knockdown lead to decreased lipid storage, as indicated by a significant drop in the lipid content and reduced expression of *Fas1* and *Fas2* (Figures 5G–J). Collectively, these results indicate that knockdown of the *JHE* and *JHEH* enhanced JH signaling, and also clearly reduced the diapause-associated phenotypes.

### Exogenous JHA Blocks Diapause by Enhancing JH Signaling

**FIGURE 6:**
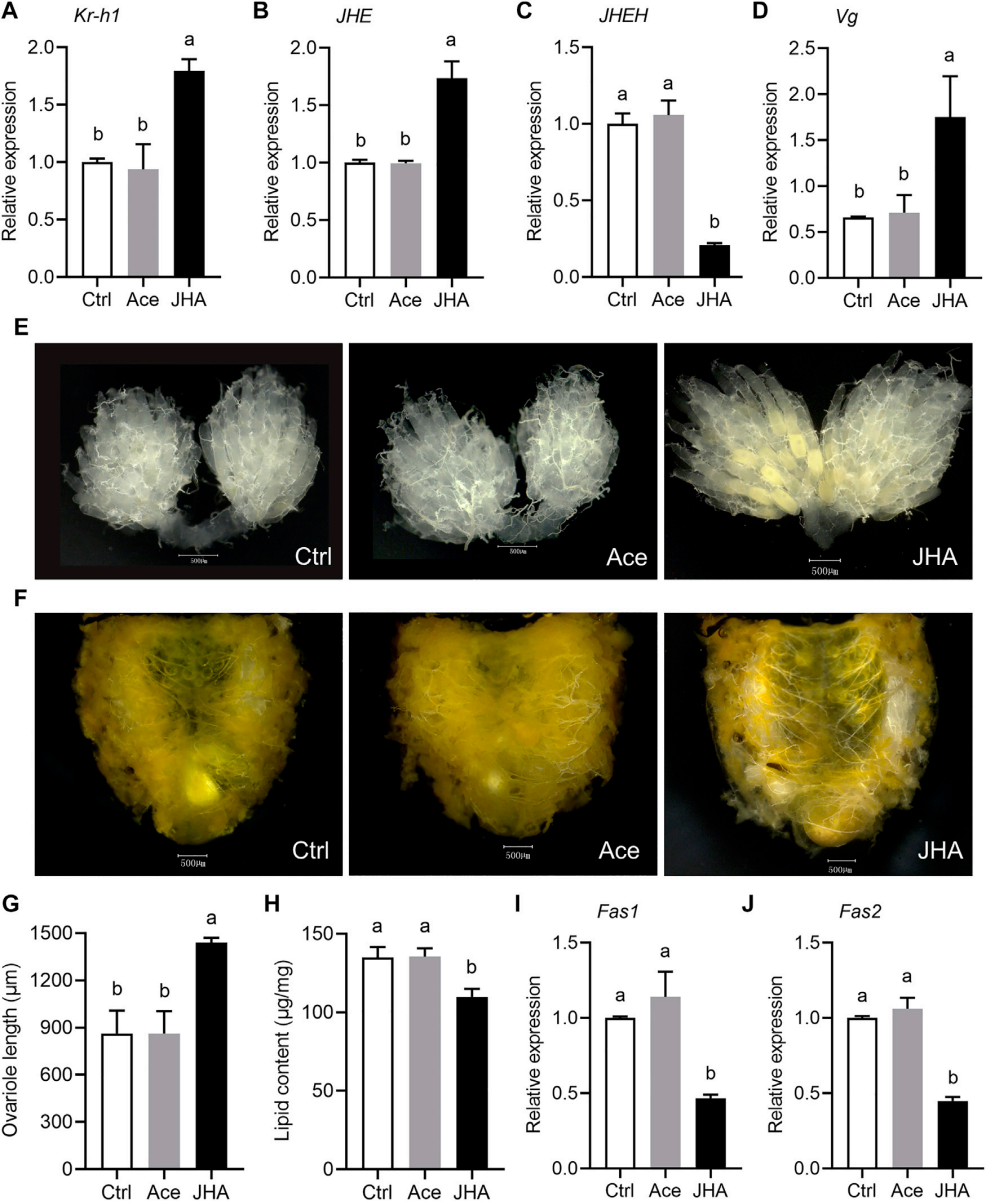
Topical application of juvenile hormone analog (JHA) methoprene to the *C. septempunctata* females during diapause preparation blocked diapause response and stimulated reproduction. The newly emerged females under diapause-inducing conditions for 2 days were treated with 100 μg methoprene (JHA), and the negative and blank controls accepted equal amounts of acetone (Ace) and double distilled water (Ctrl), respectively. Changes in mRNA abundance of JH-response gene *Kr-h1* **(A)**, *JHE* **(B)**, *JHEH* **(C)** and *Vg* **(D)**, yolk deposition **(E)**, lipid storage state **(F)**, ovariole length **(G)**, lipid content **(H)**, and expressions of *Fas1* **(I)** and *Fas2* **(J)** were measured at 11 days after treatments. The mRNA abundances of genes were expressed as fold changes compared to the blank control (Ctrl). Ovarian developmental state of females in the blank control and acetone treated control groups were photographed by a VH-Z20W zoom lens with ×100 magnification, and a ×50 magnification of camera lens was used for photographing ovary development of JHA treated females and lipid storage of females in all groups. All the values are expressed as means ± SD based on three or ten independent biological replicates. Different letters indicate significant differences (one way ANOVA followed by Tukey’s multiple comparations, *p* < 0.05). Asterisks (*) represent significant differences between the control and treatment (Student’s *t*-test; *p* < 0.05).

Topical application of JHA to the diapause-programmed females induced a significant upregulation of the early JH-response gene *Kr-h1*, suggesting that JH levels were effectively elevated (Figure 6A). Following JHA treatment, the expression level of *JHE* was significantly increased, but *JHEH* expression was reduced (Figures 6B,C). Diapause-destined females typically have tiny, inactive ovaries, ovarioles that lack vitelline, and large amounts of lipids. Compared with the control groups, JHA treated females had high levels of yolk deposits, large ovaries, and reduced lipid accumulation (Figures 6E–H). In addition, JHA treated females had higher expression of *Vg* (Figure 6D), but had lower levels of *Fas1* and *Fas2* mRNA (Figures 6I,J). Taken together, these results suggest that exogenous JHA can enhance JH signaling and reverse the diapause program. Furthermore, JH is one of the major regulators responsible for reproductive diapause in the ladybeetle, *C. septempunctata*.

## Discussion

This study explores JH regulation of diapause in females of *C. septempunctata*. Taken together, the data support our hypothesis that diapause in this species is, at least partly, due to a reduction in JH titers and that is brought about by JH degradation. Applying exogenous JHA to the diapause-destined females significantly increased JH levels and suppressed diapause entry. This suggests that JH absence is critical for diapause to occur in *C. septempunctata* females*.* Expression of both *JHE* and *JHEH* increases quickly in diapause preparation and maintain at a high level throughout diapause in the ladybeetle females. Moreover, knocking down these genes in diapause-destined females enhances JH signaling and promotes ovarian development but decreases lipid accumulation, and thus blocks diapause. These results indicate that upregulation of *JHE* and *JHEH* are essential components for generating the reproductive diapause phenotypes.

A reduction of JH is associated with diapause entry in most insect species with a reproductive diapause (Denlinger, 2002; Denlinger et al., 2012). Our results were consistent with this general observation. Treating diapause destined females of *C. septempunctata* with methoprene, a JH analog, blocked the arrest of ovarian development, suppression of vitellogenesis, and accumulation of lipids which are the hallmark features of reproductive diapause of this ladybeetle. Although JH titer was not measured in our experiments, upregulation of JH-inducible genes *Kr-h1* and *Vg* suggests that JH levels were elevated after JHA treatment, thus indirectly confirming that a much lower of JH is at least one of the major factors inducing reproductive diapause in *C. septempunctata*. This is consistent with evidence that methoprene blocks the reproductive diapause in the Asian ladybeetle *H. axyridis* (Sakurai et al., 1992; Gao et al., 2021) and the cabbage beetle *C. bowringi* (Liu et al., 2016). In addition, application of methoprene to the diapausing adults of the subspecies *Coccinella septempunctata brucki*, in which summer diapause is mainly controlled by JH, terminated diapause in both females and males (Okuda and Chinzei, 1988; Okuda, 2000). These results support our findings that JH absence is a major regulator of reproductive diapause in *C. septempunctata*.

JHE and JHEH are the primary regulatory enzymes in the degradation of JH. JHE is carboxylesterase which hydrolyzes JH to JH acid in a reversible reaction, and then JH acid can be catalyzed to an inactive JH acid diol by JHEH (Kamita and Hammock, 2010). JHEH is an epoxide hydrolase that irreversibly degrades JH to JH diol, which is then converted into either JH acid diol by JHE or to inactive JH diol phosphate by JHDK (Goodman and Cusson, 2012; Kamita et al., 2013) (Supplementary Figure S1). Several homologous *JHE* and *JHEH* genes have been cloned from coleopteran species, including *H. axyridis* (Gao et al., 2021), *Leptinotarsa decemlineata* (Lu et al., 2015), *Psacothea hilaris* (Munyiri and Ishikawa, 2007), *Tribolium castaneum* (Tsubota et al., 2010), and *C. bowringi* (Zhu et al., 2017; Guo et al., 2019). Multiple sequence alignment revealed that *JHE* and *JHEH* sequences from *C. septempunctata* that we cloned contain conserved active domains and motifs that have been identified in the corresponding proteins from other insects (Zhang et al., 2005; Kamita and Hammock, 2010; Kamita et al., 2013; Zhou K. et al., 2014; Tusun et al., 2017; Zhu et al., 2017; Guo et al., 2019). A phylogenetic tree analyses showed that CsJHE and CsJHEH are highly similar to coleopteran JHEs and JHEHs, respectively. These results suggest that CsJHE and CsJHEH are able to catalyze JH. The putative CsJHE and CsJHEH proteins both have a predicted signal peptide at the N-terminus, implying these enzymes are secreted to extracellular space. JHE is primarily synthesized in fat bodies and then secreted in the hemolymph (Kamita and Hammock, 2010), and thus the presence of the signal peptide is a diagnostic indicator of JHEs. A signal peptide has also been observed in JHEH1 from *C. bowringi* (Guo et al., 2019) and JHEH from the giant freshwater prawn *Macrobrachium rosenbergii* (Chen X. et al., 2021). However, a signal sequence is not found in *C. bowringi* JHEH2 or in JHEHs of *Bombyx mori* (Zhang et al., 2005), *Apolygus lucorum* (Tusun et al., 2017), and *T. castaneum* (Tsubota et al., 2010). These results suggest that different forms of JHEH may be active in different tissues in insects. As found in other insects, CsJHEH has a transmembrane domain at the N-terminus (Zhang et al., 2005; Wen et al., 2018), which provides evidence that CsJHEH is a microsomal epoxide hydrolase that is responsible for hydration of the epoxide (Goodman and Cusson, 2012). The enzymatic activities of JHE and JHEH in *C. septempunctata* were not examined in this study, but the upregulated expression of *Kr-h1* after silencing JHE and JHEH indirectly confirmed the potential activity of these two enzymes in JH degradation. Moreover, knockdown of *CsJHE* induced an upregulation of *CsJHEH*, and vice versa, suggesting that there is a feedback regulation in the JH degradation pathway. These results further supported the hydrolysis function of CsJHE and CsJHEH in the metabolism of JH.

Increased JH degradation is a general feature in the hormonal regulation of reproductive diapause (Vermunt et al., 1999; Guo et al., 2019; Chen H. et al., 2021; Ma et al., 2021a; Gao et al., 2021). Our observations that *JHE* and *JHEH* were substantially elevated before and during diapause and then reduced after diapause termination are consistent with results of other studies on *C. septempunctata* females (Qi et al., 2015), and with results of studies on other insects (Ma et al., 2021a). These results indicate that changes in the transcription levels of JH degradation genes are tightly associated with the process of diapause.

We think it is likely that regulation of *JHE* occurs at the transcriptional level. JHE activity in the hemolymph is positively correlated with the transcript abundance of JHE mRNA during diapause preparation in *L. decemlineata* (Vermunt et al., 1999), and we suspect there is a similar correlation in *C. septempunctata*. Interestingly, the mRNA abundance of *JHE* peaked at day 30 of diapause induction, and then declined during diapause maintenance of *C. septempunctata*. However, the peak expression of *JHEH* is later than that of *JHE*, and *JHEH* expression maintained at a high level throughout diapause. In diapausing adults of *G. daurica*, the highest expression of *JHE* was also observed at the onset of diapause. *JHEH* peaked 3 days later and then gradually decreased until the end of diapause (Ma et al., 2021a). These results imply that the expression of *JHE* and *JHEH* may regulated through different mechanisms, and it is possible that these two genes mediate JH degradation at different phases of diapause.

It is clear that *JHE* transcription is tied to JH titers, but it is not clear how they are connected in *C. septempunctata*. It is possible that JH regulates *JHE* transcription. Both JH and JH analogs, including methoprene, pyriproxyfen and fenoxycarb, increase transcription of *JHEs* in *C. bowringi* (Zhu et al., 2017), *Plutella xylostella* (Duan et al., 2016) and *B. mori* (Kamimura et al., 2007). Our results were consistent with these other studies and showed that treatment with methoprene significantly increased the expression level of *JHE* in *C. septempunctata*. In addition, a recent study on *C. bowringi* demonstrated that application of JHA elevated the expression of *JHE1* and *JHE2*, while knockdown of the JH-receptor, Methoprene-tolerant (*Met*), significantly suppressed the expression of *JHEs* (Zhu et al., 2017). Similarly, depletion of *Met* also decreased *JHE* expression in diapausing adults of *G. daurica* (Ma et al., 2021a). Together these results suggest that JH signaling regulates *JHE* transcription via the intracellular receptor *Met*. However, in diapause-destined *G. daurica* (Ma et al., 2021b) and *L. decemlineata* (Vermunt et al., 1999), JHA treatment led to a decrease in *JHE* expression and activity. The observed differences among species suggest that the regulation of *JHE* expression varies between insects, and they indicate that the question of whether *JH* signaling regulates the transcription of *JHE* in *C. septempunctata* needs further study.

The expression of *JHEH* is also connected to JH titers. In diapause-destined females of *C. septempunctata JHEH* transcription decreased after JHA treatment. Our results are similar to those from studies on the transcript levels of *JHEH* in *G. daurica* (Ma et al., 2021b) and *JHEH2* in *C. bowringi* (Guo et al., 2019) following the addition of JHA. The downregulation of the JH-*Met*-*Kr-h1* signaling pathway induces the upregulation of *JHEH2*, but not *JHEH1*, during diapause preparation in females of *C. bowringi* (Guo et al., 2019). However, depletion of *Met* had no significant influence on the expression of *JHEH* in *G. daurica* (Ma et al., 2021a). Given the different homologs of *JHEH* reported in these studies, more evidence is needed to identify the upstream signals that regulates the expression of *JHEHs*.

The results we present here provide evidence that elevated expression of *JHE* and *JHEH* in diapausing *C. septempunctata* females can promote reproductive diapause by reducing JH level. Knocking down *JHE* or *JHEH* in diapause-destined ladybeetle females led to significantly upregulated expression of *Kr-h1*, which indicates that JH titer increased following RNA interference of these two genes. Moreover, silencing either *JHE* or *JHEH* during diapause preparation of *C. septempunctata* promoted both ovarian development and yolk deposition, and prevented lipid accumulation. Our conclusions are supported by evidence from studies on *H. axyridis* (Gao et al., 2021) and *C. bowringi* (Guo et al., 2019). For both of these species, reduced expression of *JHEHs* in diapause-destined females lead to increased JH signaling and *Vg* expression, as well as reduced fat storage and downregulation of genes involved in lipogenesis, lipolysis and stress-tolerance. However, knockdown of *JHEHs* in these two species failed to reverse the arrested ovarian development. It is possible that slightly different responses to *JHEH* knockdown is due to the amount of dsRNA used for gene knockdown. Indeed, the authors of these studies proposed that the effect of gene knockdown on diapause is dose dependent. An alternative explanation is that there may be species differences in the role of *JHEH* in regulating diapause. This second alternative seems likely. In contrast with our results, silencing *JHE1* during diapause preparation of *H. axyridis* females did not affect either JH signaling or alter diapause-related traits. These results suggest that *JHE1* may not contribute to reproductive diapause in *H. axyridis* (Gao et al., 2021). Given the important role of *JHEs* and *JHEHs* in regulating reproductive diapause, these genes represent potential targets to manipulate the program of adult diapause, thus facilitating the mass production and application of beneficial insects.

## Conclusion

Our experiments reveal that JH inhibition is likely to be the primary contributor of reproductive diapause in the ladybeetle *C. septempunctata*, as the diapause-associated phenotypes can be reversed by application of JHA. Knocking down of JH degradation genes, *JHE* and *JHEH*, in females preparing to enter diapause suppressed key features of reproductive diapause phenotypes, including arrested ovarian development and lipid accumulation, evidencing a critical role of JH degradation in the induction of reproductive diapause of *C. septempunctata*. The high expression of JH degradation genes during diapause maintenance suggests that reducing JH metabolism may also play a major role in maintaining diapause. Functions of JH degradation pathways and the related upstream regulators during reproductive diapause should be further explored. The results we obtained with *C. septempunctata* suggest the possibility that manipulating diapause by exogenous JHA or targeting JH signaling could be used to facilitate the commercial production of biological control agents. Simple methods for increasing shelf-life or quickly terminating diapause of predators may be attractive for mass rearing and effective release of biological control agents in biological control programs.

## Data Availability

The datasets presented in this study can be found in online repositories. The names of the repository/repositories and accession number(s) can be found in the article/Supplementary Material.

## Footnotes

1
http://web.expasy.org/translate/.

2
https://www.ncbi.nlm.nih.gov/cdd.

3
http://www.cbs.dtu.dk/services/SignalP/.

4
http://www.cbs.dtu.dk/services/TMHMM/.

5
https://www.ncbi.nlm.nih.gov/.

6
https://espript.ibcp.fr/ESPript/ESPript/.
