# Supplementary material for: Enhanced Degradation of Juvenile Hormone Promotes Reproductive Diapause in the Predatory Ladybeetle Coccinella Septempunctata
Source: Front Physiol. 2022 Apr 29;13:877153. doi: 10.3389/fphys.2022.877153 (PMC9099232; doi:10.3389/fphys.2022.877153)
Supplement: Supplementary file 1 [file Table1.DOCX]

**Table S1** Sequences of primers used for cDNA cloning, quantitative real-time PCR (qRT-PCR), and double-stranded RNA (dsRNA) synthesis.

| **Primer name** | **Primer sequence** |
| --- | --- |
| JHE-f | TTCACAAAAACTAATCCTGCCACT |
| JHE-r | TATGAAATGGTGGGTTTCCATATT |
| JHEH-f1 | GTGCTAAACATACTTACGGTATTTCATC |
| JHEH-r1 | CCAGCCGTGGATGAGCAA |
| JHEH-f2 | TGATTTTGGAGTCTGGGTATTTACA |
| JHEH-r2 | ATGACGTTATTGTACCAGTTACCCA |
| RACE-JHE-f1 | GGCATTAGAGGTGTGTCTCACGAGGAAG |
| RACE-JHE-f2 | ACAGATTATGTGGTCAGGGAATGGATGTTA |
| RACE-JHE-r1 | AGTCCGTAATTGCCAGGTGATGCCA |
| RACE-JHE-r2 | GATGCCAAATCCTCGGTGCTCAAGA |
| RACE-JHEH-f1 | GTTCGAGATTTGCACACGACATAGTGTACG |
| RACE-JHEH-f2 | CACCTACCGCCCTGTTGGAGGATAA |
| RACE-JHEH-r1 | TTTGAAAGGTCTTATCGTGACATCGTCTACA |
| RACE-JHEH-r2 | CAAGTGTCTTCTAATTTCGGGATAGGTGGA |
| qJHE-f | GACCAAAACCTTGCCCTACG |
| qJHE-r | CCACAAACATAGAGCACTTCCG |
| qJHEH-f | CTTTCAAAATCGCCGTTCC |
| qJHEH-r | AAGTCCAGCACTGATTTCGTG |
| qVg-f | AAACACTCCAATGCGGTC |
| qVg-r | GAGAATGATGTAGGCAGCG |
| qFas1-f | CCGTAGTCTGCCAAACATCC |
| qFas1-r | AAATCCTCAACAGCAACGACTC |
| qFas2-f | TTTGGCGATAGAACATAGAGCA |
| qFas2-r | AGCCCACGGACAGGAAC |
| qKr-h1-f | CAAGTGTGAGGTCTGTTCTAGGG |
| qKr-h1-r | GGCATACTTGACAGACGTAAGG |
| q18S-f | GGACCTCGATTCTATTTTGTTGG |
| q18S-r | TCGCTTCTGTCCGTCTTGC |
| qActin-f | GATTCGCCATCCAGGACATCTC |
| qActin-r | TCCTTGCTCAGCTTGTTGTAGTC |
| dsJHE-f | GCGTAATACGACTCACTATAGGGATGGGAGCAAACAAGGAT |
| dsJHE-r | GCGTAATACGACTCACTATAGGGGGCGACACCGAGAAGATA |
| dsJHEH-f | GCGTAATACGACTCACTATAGGGCGAAGAAGATTGAGGGACTC |
| dsJHEH-r | GCGTAATACGACTCACTATAGGGTGGTTTGGTAGCCTGTAGATG |
| dsGFP-f | TAATACGACTCACTATAGGGCACAAGTTCAGCGTGTCCG |
| dsGFP-r | TAATACGACTCACTATAGGGAGTTCACCTTGATGCCGTTC |
