# Supplementary figures and images for: Enhanced Degradation of Juvenile Hormone Promotes Reproductive Diapause in the Predatory Ladybeetle Coccinella Septempunctata
Source: Front Physiol. 2022 Apr 29;13:877153. doi: 10.3389/fphys.2022.877153 (PMC9099232; doi:10.3389/fphys.2022.877153)

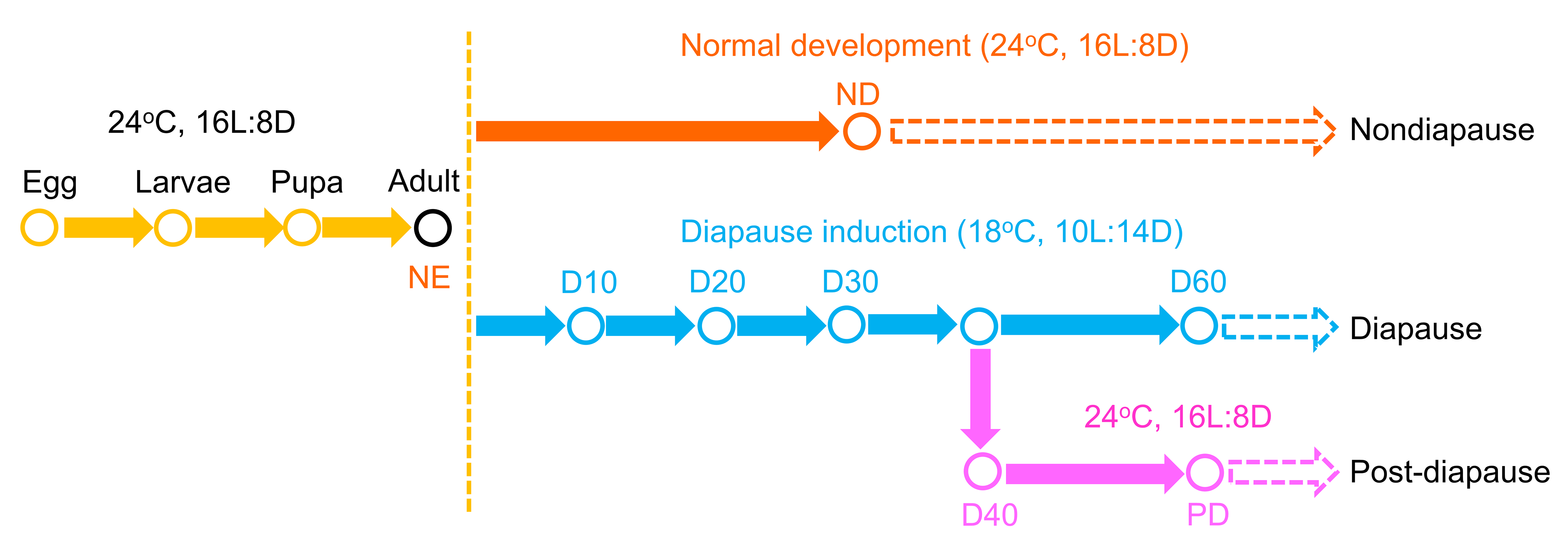

Supplement: Supplementary file 2 [file Image2.TIF]

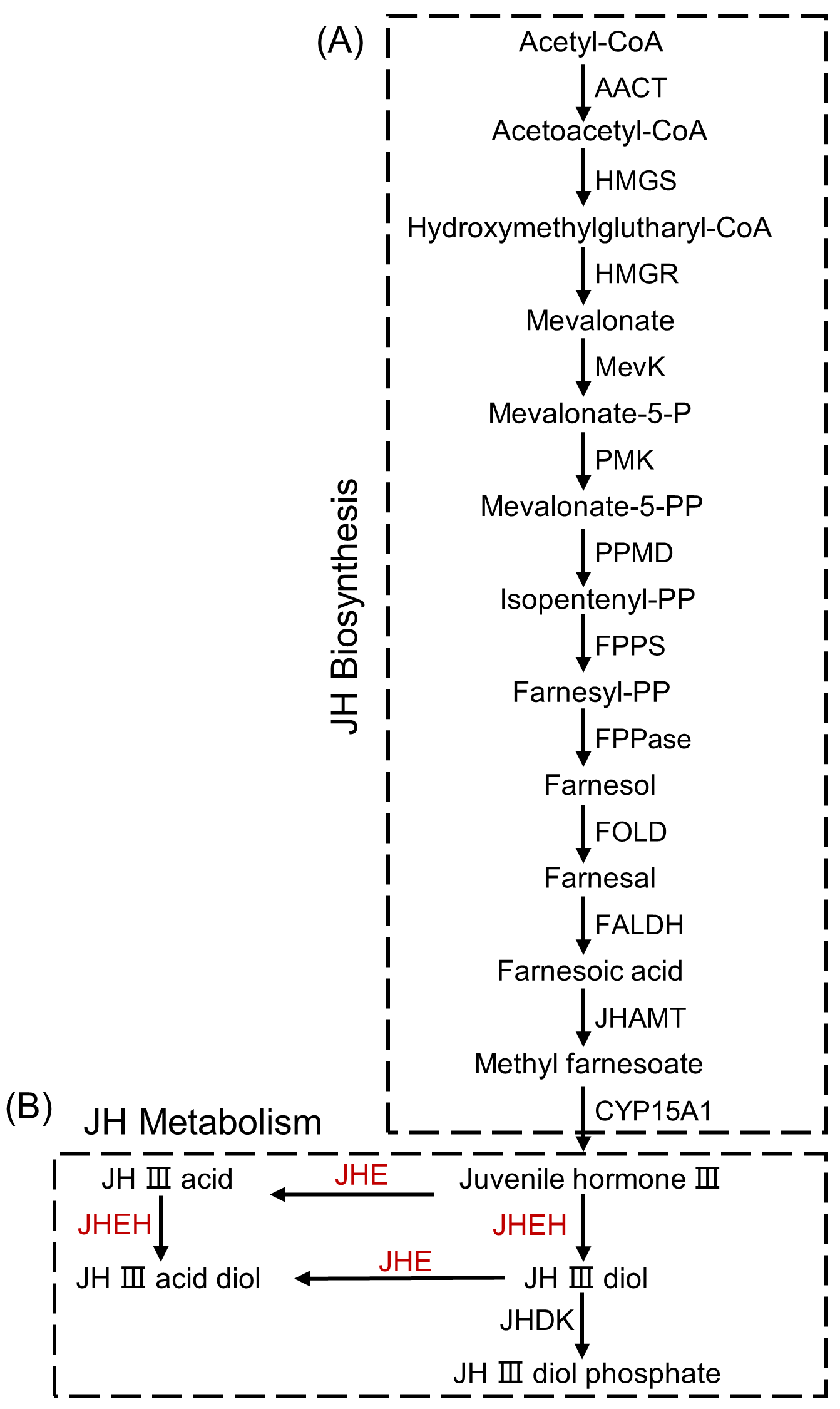

Supplement: Supplementary file 3 [file Image1.tif]
